# Behavioral determinants of hand hygiene compliance in a pediatric intensive care unit from Brazil

**DOI:** 10.1186/2197-425X-3-S1-A721

**Published:** 2015-10-01

**Authors:** ASC Belela-Anacleto, DM Kusahara, MA Peterlini, M Pedreira

**Affiliations:** Federal University of São Paulo, Pediatric Nursing, São Paulo, Brazil

## Introduction

Despite evidences of hand hygiene (HH) on reduction of health care associated infection levels, HH compliance among healthcare professionals is described as unacceptably low worldwide. Beyond lack of knowledge, difficulties related to staff motivation to HH compliance can be influenced by behavioral determinants.

## Objectives

To identify predictors of intention to perform the behavior 'HH during patient care in the pediatric intensive care unit (PICU)'.

## Methods

Descriptive study performed in a nine bed PICU at a university hospital in São Paulo, Brazil. Based on Theory of Planned Behavior (TPB) foundations and HH characteristics, an instrument was designed and validated (Delphi technique/ Cronbach's alpha coefficient). The theory suggests that intention is the best single predictor of behavior and represents the readiness for its performance. Basically, individuals are prone to perform a behavior because they intend to do so. Intention is based on a particular combination of attitudinal, normative and control considerations. The instrument comprised demographic variables and TPB constructs: intention (five items), attitude (six items), perceived social pressure (PSP) (six items) and perceived behavior control (six items). Attitude was measured by six semantic differential scales constructed with bipolar adjectives. The others constructs were measured by Likert scales, all scores ranging from one to seven, and professionals were asked to indicate their opinion or judgment on the questions or assertions presented. Each construct was considered as an independent variable and scores were obtained by the arithmetic mean of the constructs' scores. Regression analysis was used to estimate the relationships among TPB determinant constructs and the intention to perform the behavior.

## Results

The global analysis of PICU staff behavioral determinants of HH compliance revealed that in 21 of the 23 items related to the TPB constructs, it was identified a mean value greater than or equal to 5.0, showing predisposition of the group to perform ´HH during patient care in the PICU´. Multivariate regression analysis revealed that PSP (p = 0.026) characterizes a determinant factor of intention to perform the behavior, unlike the other variables (attitude p = 0.452; perceived behavior control p = 0.540).

## Conclusions

Perceived social pressure was the determinant factor of the intention to perform HH during patient care in PICU. Conceptually, it refers to people beliefs that important individuals or groups would approve or disapprove their performing of a behavior and that these referents themselves perform or not this behavior. Social influences had a direct impact on HH compliance behavior of the studied PICU staff and should be addressed when designing HH promotion interventions.

